# Diaqua­bis(4-methyl­benzoato-κ*O*)bis­(nicotinamide-κ*N* ^1^)manganese(II)

**DOI:** 10.1107/S1600536810011815

**Published:** 2010-04-10

**Authors:** Hacali Necefoğlu, Efdal Çimen, Barış Tercan, Hakan Dal, Tuncer Hökelek

**Affiliations:** a Department of Chemistry, Kafkas University, 36100 Kars, Turkey; b Department of Physics, Karabük University, 78050 Karabük, Turkey; c Department of Chemistry, Faculty of Science, Anadolu University, 26470 Yenibağlar, Eskişehir, Turkey; d Department of Physics, Hacettepe University, 06800 Beytepe, Ankara, Turkey

## Abstract

In the mononuclear title complex, [Mn(C_8_H_7_O_2_)_2_(C_6_H_6_N_2_O)_2_(H_2_O)_2_], the Mn^II^ ion is located on a crystallographic inversion center. The asymmetric unit contains one 4-methyl­benzoate anion, one nicotinamide (NA) ligand and one coordinated water mol­ecule. The four O atoms in the equatorial plane around the Mn^II^ ion form a slightly distorted square-planar arrangement, while the slightly distorted octa­hedral coordination is completed by the two pyridine N atoms of the NA ligands in the axial positions. The dihedral angle between the carboxyl­ate group and the attached benzene ring is 9.01 (7)°, while the pyridine and benzene rings are oriented at a dihedral angle of 42.44 (5)°. In the crystal structure, inter­molecular O—H⋯O, N—H⋯O and C—H⋯O hydrogen bonds, and O—H⋯π and C—H⋯π inter­actions link the mol­ecules into a two-dimensional network parallel to (001).

## Related literature

For niacin, see: Krishnamachari (1974 ▶), and for the nicotinic acid derivative *N*,*N*-diethyl­nicotinamide, see: Bigoli *et al.* (1972 ▶). For related structures, see: Hökelek *et al.* (1996 ▶, 2009*a* ▶,*b* ▶,*c* ▶); Hökelek & Necefoğlu (1998 ▶); Necefoğlu *et al.* (2010 ▶).
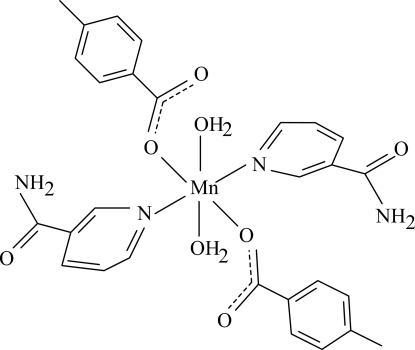


## Experimental

### 

#### Crystal data

[Mn(C_8_H_7_O_2_)_2_(C_6_H_6_N_2_O)_2_(H_2_O)_2_]
*M* *_r_* = 605.51
Triclinic, 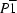

*a* = 7.3289 (2) Å
*b* = 10.1768 (3) Å
*c* = 10.6292 (3) Å
α = 66.852 (2)°
β = 78.232 (4)°
γ = 70.206 (3)°
*V* = 683.58 (4) Å^3^
*Z* = 1
Mo *K*α radiation
μ = 0.54 mm^−1^
*T* = 100 K
0.38 × 0.25 × 0.19 mm

#### Data collection

Bruker Kappa APEXII CCD area-detector diffractometer
Absorption correction: multi-scan (*SADABS*; Bruker, 2005 ▶) *T*_min_ = 0.649, *T*_max_ = 0.698
11619 measured reflections
3297 independent reflections
3022 reflections with *I* > 2σ(*I*)
*R*_int_ = 0.028

#### Refinement

*R*[*F* ^2^ > 2σ(*F* ^2^)] = 0.035
*wR*(*F* ^2^) = 0.099
*S* = 1.08
3297 reflections
204 parameters
3 restraints
H atoms treated by a mixture of independent and constrained refinement
Δρ_max_ = 0.73 e Å^−3^
Δρ_min_ = −0.38 e Å^−3^

### 

Data collection: *APEX2* (Bruker, 2007 ▶); cell refinement: *SAINT* (Bruker, 2007 ▶); data reduction: *SAINT*; program(s) used to solve structure: *SHELXS97* (Sheldrick, 2008 ▶); program(s) used to refine structure: *SHELXL97* (Sheldrick, 2008 ▶); molecular graphics: *ORTEP-3 for Windows* (Farrugia, 1997 ▶); software used to prepare material for publication: *WinGX* (Farrugia, 1999 ▶) and *PLATON* (Spek, 2009 ▶).

## Supplementary Material

Crystal structure: contains datablocks I, global. DOI: 10.1107/S1600536810011815/ci5069sup1.cif

Structure factors: contains datablocks I. DOI: 10.1107/S1600536810011815/ci5069Isup2.hkl

Additional supplementary materials:  crystallographic information; 3D view; checkCIF report

## Figures and Tables

**Table 1:** Selected bond lengths (Å)

Mn1—O2	2.1036 (11)
Mn1—O4	2.1924 (12)
Mn1—N1	2.2947 (13)

**Table 2:** Hydrogen-bond geometry (Å, °) *Cg*1 and *Cg*2 are the centroids of the C2-C7 andN1/C9-C13 rings, respectively.

*D*—H⋯*A*	*D*—H	H⋯*A*	*D*⋯*A*	*D*—H⋯*A*
N2—H21⋯O1^i^	0.88 (3)	1.97 (3)	2.8456 (19)	174 (2)
N2—H22⋯O3^ii^	0.86 (3)	2.09 (3)	2.952 (2)	172 (3)
O4—H42⋯O3^iii^	0.90 (3)	1.83 (3)	2.7071 (19)	164 (3)
C11—H11⋯O1^i^	0.93	2.33	3.200 (2)	156
O4—H41⋯*Cg*1^iv^	0.91 (2)	2.33 (2)	3.141 (2)	149 (3)
C4—H4⋯*Cg*2^iv^	0.93	2.80	3.490 (2)	132

Symmetry codes: (i) 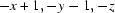; (ii) 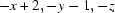; (iii) 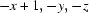; (iv) 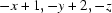.

**Fig. 1.**
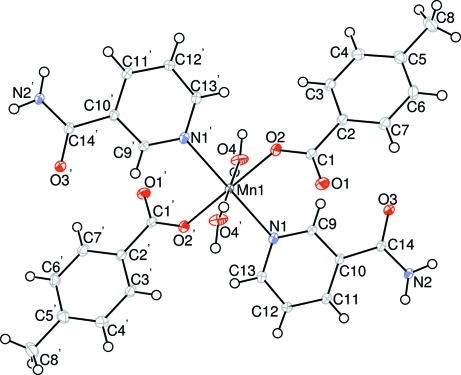
The molecular structure of the title compound, with the atom-numbering scheme. Displacement ellipsoids are drawn at the 50% probability level. Primed atoms are generated by the symmetry operator:(') -x, -y, - z.

## supplementary crystallographic information

### Comment

As a part of our ongoing investigation on transition metal complexes of nicotinamide (NA), one form of niacin (Krishnamachari, 1974), and/or the nicotinic acid derivative *N*,*N*-diethylnicotinamide (DENA), an important respiratory stimulant (Bigoli *et al.*, 1972), the title compound was synthesized and its crystal structure is reported herein.

The title compound, (I), is a centrosymmetric mononuclear complex, consisting of two nicotinamide (NA) and two 4-methylbenzoate (PMB) ligands and two coordinated water molecules; the Mn^II^ ion lies on a centre of symmetry (Fig. 1). The crystal structures of some PMB and/or NA complexes of Cu^II^, Co^II^, Ni^II^, Mn^II^ and Zn^II^ ions, [Cu(C_7_H_5_O_2_)_2_(C_10_H_14_N_2_O)_2_], (II) (Hökelek *et al.*, 1996), [Co(C_6_H_6_N_2_O)_2_(C_7_H_4_NO_4_)_2_(H_2_O)_2_], (III) (Hökelek & Necefoğlu, 1998), [Ni(C_7_H_4_ClO_2_)_2_(C_6_H_6_N_2_O)_2_(H_2_O)_2_], (IV) (Hökelek *et al.*, 2009a), [Ni(C_8_H_7_O_2_)_2_(C_6_H_6_N_2_O)_2_(H_2_O)_2_], (V) (Necefoğlu *et al.*, 2010), [Mn(C_7_H_4_ClO_2_)_2_(C_10_H_14_N_2_O)_2_(H_2_O)_2_], (VI) (Hökelek *et al.*, 2009b) and [Zn(C_7_H_4_BrO_2_)_2_(C_6_H_6_N_2_O)_2_(H_2_O)_2_], (VII) (Hökelek *et al.*, 2009c) have also been reported. In (II), the two benzoate ions are coordinated to the Cu atom as bidentate ligands, while in other structures all ligands being monodentate.

In the title compound, all ligands are monodentate. The four O atoms (O2, O4, and the symmetry-related atoms, O2', O4') in the equatorial plane around the Mn^II^ atom form a slightly distorted square-planar arrangement, while the slightly distorted octahedral coordination is completed by the two N atoms of the NA ligands (N1, N1') in the axial positions (Fig. 1). The near equality of the C1—O1 [1.2466 (19) Å] and C1—O2 [1.2690 (18) Å] bonds in the carboxylate group indicates a delocalized bonding arrangement, rather than localized single and double bonds. The average Mn—O bond length is 2.148 (1) Å (Table 1). The dihedral angle between the planar carboxylate group (O1/O2/C1) and the benzene ring A (C2—C7) is 9.01 (7)°, while that between rings A and B (N1/C9—C13) is 42.44 (5)°.

In the crystal structure, intermolecular O—H···O, N—H···O and C—H···O hydrogen bonds (Table 2) link the molecules into a two-dimensional network parallel to the (001). Weak O—H···π and C—H···π interactions involving the pyridine and benzene rings are also observed (Table 2).

### Experimental

The title compound was prepared by the reaction of MnSO_4_.H_2_O (0.84 g, 5 mmol) in H_2_O (10 ml) and NA (1.22 g, 10 mmol) in H_2_O (10 ml) with sodium 4-methylbenzoate (1.58 g, 10 mmol) in H_2_O (150 ml). The mixture was filtered and set aside to crystallize at ambient temperature for one month, giving colourless single crystals.

### Refinement

H atoms of the NH_2_ group (H21 and H22) and water molecules (H41 and H42) were located in a difference Fourier map and refined isotropically; the water H atoms were refined with O–H and H···H distance restraints. The remaining H atoms were positioned geometrically with C–H = 0.93 and 0.96 Å, for aromatic and methyl H atoms, respectively, and constrained to ride on their parent atoms, with U_iso_(H) = xU_eq_(C), where x = 1.5 for methyl H and x = 1.2 for aromatic H atoms.

### Crystal data

**Table:**

[Mn(C_8_H_7_O_2_)_2_(C_6_H_6_N_2_O)_2_(H_2_O)_2_]	*Z* = 1
*M**_r_* = 605.51	*F*(000) = 315
Triclinic, *P*1	*D*_x_ = 1.471 Mg m^−^^3^
Hall symbol: -P 1	Mo *K*α radiation, λ = 0.71073 Å
*a* = 7.3289 (2) Å	Cell parameters from 8087 reflections
*b* = 10.1768 (3) Å	θ = 2.5–28.4°
*c* = 10.6292 (3) Å	µ = 0.54 mm^−^^1^
α = 66.852 (2)°	*T* = 100 K
β = 78.232 (4)°	Block, colourless
γ = 70.206 (3)°	0.38 × 0.25 × 0.19 mm
*V* = 683.58 (4) Å^3^	

### Data collection

**Table:**

Bruker Kappa APEXII CCD area-detector diffractometer	3297 independent reflections
Radiation source: fine-focus sealed tube	3022 reflections with *I* > 2σ(*I*)
graphite	*R*_int_ = 0.028
φ and ω scans	θ_max_ = 28.4°, θ_min_ = 2.1°
Absorption correction: multi-scan (*SADABS*; Bruker, 2005)	*h* = −9→9
*T*_min_ = 0.649, *T*_max_ = 0.698	*k* = −11→13
11619 measured reflections	*l* = −14→14

### Refinement

**Table:**

Refinement on *F*^2^	Primary atom site location: structure-invariant direct methods
Least-squares matrix: full	Secondary atom site location: difference Fourier map
*R*[*F*^2^ > 2σ(*F*^2^)] = 0.035	Hydrogen site location: inferred from neighbouring sites
*wR*(*F*^2^) = 0.099	H atoms treated by a mixture of independent and constrained refinement
*S* = 1.08	*w* = 1/[σ^2^(*F*_o_^2^) + (0.058*P*)^2^ + 0.3059*P*] where *P* = (*F*_o_^2^ + 2*F*_c_^2^)/3
3297 reflections	(Δ/σ)_max_ = 0.001
204 parameters	Δρ_max_ = 0.73 e Å^−^^3^
3 restraints	Δρ_min_ = −0.38 e Å^−^^3^

### Special details

**Table:**

Geometry. All esds (except the esd in the dihedral angle between two l.s. planes) are estimated using the full covariance matrix. The cell esds are taken into account individually in the estimation of esds in distances, angles and torsion angles; correlations between esds in cell parameters are only used when they are defined by crystal symmetry. An approximate (isotropic) treatment of cell esds is used for estimating esds involving l.s. planes.
Refinement. Refinement of *F*^2^ against ALL reflections. The weighted *R*-factor wR and goodness of fit *S* are based on *F*^2^, conventional *R*-factors *R* are based on *F*, with *F* set to zero for negative *F*^2^. The threshold expression of *F*^2^ > σ(*F*^2^) is used only for calculating *R*-factors(gt) etc. and is not relevant to the choice of reflections for refinement. *R*-factors based on *F*^2^ are statistically about twice as large as those based on *F*, and *R*- factors based on ALL data will be even larger.

### Fractional atomic coordinates and isotropic or equivalent isotropic displacement parameters (Å^2^)

**Table:**

	*x*	*y*	*z*	*U*_iso_*/*U*_eq_	
Mn1	0.0000	0.0000	0.0000	0.01276 (11)	
O1	0.24237 (17)	−0.33733 (12)	0.25988 (12)	0.0207 (2)	
O2	0.16077 (17)	−0.08763 (12)	0.17238 (12)	0.0201 (2)	
O3	0.78404 (16)	−0.34130 (13)	−0.01469 (13)	0.0210 (3)	
O4	0.18323 (19)	0.15102 (14)	−0.09400 (13)	0.0232 (3)	
H41	0.208 (4)	0.190 (3)	−0.1860 (17)	0.064 (9)*	
H42	0.218 (4)	0.205 (3)	−0.058 (2)	0.042 (7)*	
N1	0.21166 (18)	−0.15932 (14)	−0.10409 (13)	0.0143 (3)	
N2	0.84692 (19)	−0.50868 (14)	−0.11834 (14)	0.0167 (3)	
H21	0.810 (3)	−0.555 (3)	−0.159 (2)	0.028 (5)*	
H22	0.958 (4)	−0.545 (3)	−0.084 (2)	0.032 (6)*	
C1	0.2620 (2)	−0.21470 (17)	0.24423 (15)	0.0151 (3)	
C2	0.4196 (2)	−0.21314 (16)	0.31505 (15)	0.0145 (3)	
C3	0.4331 (2)	−0.07942 (16)	0.31453 (15)	0.0164 (3)	
H3	0.3426	0.0099	0.2714	0.020*	
C4	0.5805 (2)	−0.07856 (18)	0.37770 (16)	0.0181 (3)	
H4	0.5871	0.0115	0.3767	0.022*	
C5	0.7191 (2)	−0.21079 (19)	0.44279 (16)	0.0190 (3)	
C6	0.7063 (2)	−0.34413 (18)	0.44183 (16)	0.0203 (3)	
H6	0.7979	−0.4333	0.4839	0.024*	
C7	0.5589 (2)	−0.34546 (17)	0.37902 (16)	0.0177 (3)	
H7	0.5528	−0.4355	0.3795	0.021*	
C8	0.8783 (3)	−0.2097 (2)	0.51214 (18)	0.0257 (4)	
H8A	0.8947	−0.1115	0.4763	0.039*	
H8B	0.9977	−0.2797	0.4951	0.039*	
H8C	0.8437	−0.2371	0.6092	0.039*	
C9	0.3975 (2)	−0.22336 (16)	−0.07444 (15)	0.0132 (3)	
H9	0.4418	−0.1971	−0.0142	0.016*	
C10	0.5280 (2)	−0.32707 (16)	−0.12893 (15)	0.0134 (3)	
C11	0.4623 (2)	−0.36415 (17)	−0.22143 (16)	0.0170 (3)	
H11	0.5453	−0.4328	−0.2602	0.020*	
C12	0.2710 (2)	−0.29647 (18)	−0.25420 (17)	0.0198 (3)	
H12	0.2239	−0.3182	−0.3164	0.024*	
C13	0.1508 (2)	−0.19621 (17)	−0.19343 (16)	0.0172 (3)	
H13	0.0221	−0.1523	−0.2153	0.021*	
C14	0.7300 (2)	−0.39401 (16)	−0.08365 (15)	0.0142 (3)	

### Atomic displacement parameters (Å^2^)

**Table:**

	*U*^11^	*U*^22^	*U*^33^	*U*^12^	*U*^13^	*U*^23^
Mn1	0.01065 (17)	0.01054 (16)	0.01760 (17)	0.00071 (11)	−0.00334 (11)	−0.00758 (12)
O1	0.0252 (6)	0.0149 (5)	0.0241 (6)	−0.0030 (4)	−0.0062 (5)	−0.0092 (5)
O2	0.0216 (6)	0.0138 (5)	0.0243 (6)	0.0035 (4)	−0.0106 (5)	−0.0092 (4)
O3	0.0160 (5)	0.0184 (5)	0.0343 (6)	0.0021 (4)	−0.0096 (5)	−0.0174 (5)
O4	0.0308 (7)	0.0236 (6)	0.0233 (6)	−0.0146 (5)	0.0056 (5)	−0.0147 (5)
N1	0.0126 (6)	0.0117 (6)	0.0177 (6)	−0.0003 (4)	−0.0030 (5)	−0.0061 (5)
N2	0.0120 (6)	0.0146 (6)	0.0258 (7)	0.0020 (5)	−0.0057 (5)	−0.0125 (5)
C1	0.0149 (7)	0.0150 (7)	0.0155 (7)	0.0004 (5)	−0.0019 (5)	−0.0091 (6)
C2	0.0147 (7)	0.0137 (7)	0.0140 (6)	−0.0008 (5)	−0.0017 (5)	−0.0061 (5)
C3	0.0175 (7)	0.0129 (7)	0.0164 (7)	−0.0004 (5)	−0.0029 (6)	−0.0055 (6)
C4	0.0209 (8)	0.0181 (7)	0.0173 (7)	−0.0059 (6)	−0.0014 (6)	−0.0080 (6)
C5	0.0170 (7)	0.0245 (8)	0.0158 (7)	−0.0045 (6)	−0.0017 (6)	−0.0083 (6)
C6	0.0182 (7)	0.0177 (7)	0.0196 (7)	0.0026 (6)	−0.0066 (6)	−0.0053 (6)
C7	0.0203 (7)	0.0124 (7)	0.0186 (7)	−0.0006 (5)	−0.0041 (6)	−0.0056 (6)
C8	0.0199 (8)	0.0352 (10)	0.0240 (8)	−0.0067 (7)	−0.0061 (6)	−0.0114 (7)
C9	0.0137 (7)	0.0105 (6)	0.0160 (6)	−0.0017 (5)	−0.0027 (5)	−0.0060 (5)
C10	0.0118 (7)	0.0101 (6)	0.0174 (7)	−0.0007 (5)	−0.0031 (5)	−0.0051 (5)
C11	0.0146 (7)	0.0149 (7)	0.0224 (7)	0.0015 (5)	−0.0042 (6)	−0.0110 (6)
C12	0.0181 (8)	0.0194 (7)	0.0258 (8)	0.0016 (6)	−0.0092 (6)	−0.0142 (7)
C13	0.0137 (7)	0.0150 (7)	0.0229 (7)	0.0008 (5)	−0.0059 (6)	−0.0087 (6)
C14	0.0127 (7)	0.0113 (6)	0.0184 (7)	−0.0007 (5)	−0.0033 (5)	−0.0063 (5)

### Geometric parameters (Å, °)

**Table:**

Mn1—O2	2.1036 (11)	C4—C3	1.387 (2)
Mn1—O2^i^	2.1036 (11)	C4—C5	1.396 (2)
Mn1—O4	2.1924 (12)	C4—H4	0.93
Mn1—O4^i^	2.1924 (12)	C5—C8	1.507 (2)
Mn1—N1	2.2947 (13)	C6—C5	1.396 (2)
Mn1—N1^i^	2.2947 (13)	C6—C7	1.387 (2)
O1—C1	1.2466 (19)	C6—H6	0.93
O2—C1	1.2690 (18)	C7—H7	0.93
O3—C14	1.2464 (18)	C8—H8A	0.96
O4—H41	0.905 (16)	C8—H8B	0.96
O4—H42	0.906 (16)	C8—H8C	0.96
N1—C9	1.3386 (18)	C9—C10	1.391 (2)
N1—C13	1.345 (2)	C9—H9	0.93
N2—C14	1.3283 (19)	C11—C10	1.396 (2)
N2—H21	0.89 (2)	C11—C12	1.385 (2)
N2—H22	0.86 (2)	C11—H11	0.93
C1—C2	1.509 (2)	C12—H12	0.93
C2—C3	1.395 (2)	C13—C12	1.383 (2)
C2—C7	1.397 (2)	C13—H13	0.93
C3—H3	0.93	C14—C10	1.4956 (19)
			
O2—Mn1—O2^i^	180.00 (4)	C3—C4—H4	119.5
O2—Mn1—O4	86.18 (5)	C5—C4—H4	119.5
O2^i^—Mn1—O4	93.82 (5)	C4—C5—C8	120.94 (15)
O2—Mn1—O4^i^	93.82 (5)	C6—C5—C4	118.20 (14)
O2^i^—Mn1—O4^i^	86.18 (5)	C6—C5—C8	120.86 (15)
O2—Mn1—N1^i^	86.02 (4)	C5—C6—H6	119.5
O2^i^—Mn1—N1^i^	93.98 (4)	C7—C6—C5	120.91 (14)
O2—Mn1—N1	93.98 (4)	C7—C6—H6	119.5
O2^i^—Mn1—N1	86.02 (4)	C2—C7—H7	119.6
O4—Mn1—O4^i^	180.00 (5)	C6—C7—C2	120.74 (14)
O4—Mn1—N1^i^	91.32 (4)	C6—C7—H7	119.6
O4^i^—Mn1—N1^i^	88.68 (4)	C5—C8—H8A	109.5
O4—Mn1—N1	88.68 (4)	C5—C8—H8B	109.5
O4^i^—Mn1—N1	91.32 (4)	C5—C8—H8C	109.5
N1^i^—Mn1—N1	180.00 (9)	H8A—C8—H8B	109.5
C1—O2—Mn1	136.86 (10)	H8A—C8—H8C	109.5
Mn1—O4—H41	122.2 (18)	H8B—C8—H8C	109.5
Mn1—O4—H42	129.2 (16)	N1—C9—C10	123.52 (13)
H42—O4—H41	107 (2)	N1—C9—H9	118.2
C9—N1—Mn1	121.52 (10)	C10—C9—H9	118.2
C9—N1—C13	117.56 (13)	C9—C10—C11	118.15 (13)
C13—N1—Mn1	120.88 (10)	C9—C10—C14	118.12 (13)
C14—N2—H21	124.4 (15)	C11—C10—C14	123.72 (13)
C14—N2—H22	113.3 (16)	C10—C11—H11	120.7
H21—N2—H22	121 (2)	C12—C11—C10	118.59 (14)
O1—C1—O2	125.89 (14)	C12—C11—H11	120.7
O1—C1—C2	118.59 (13)	C11—C12—H12	120.4
O2—C1—C2	115.51 (13)	C13—C12—C11	119.26 (14)
C3—C2—C1	120.90 (13)	C13—C12—H12	120.4
C3—C2—C7	118.51 (14)	N1—C13—C12	122.90 (14)
C7—C2—C1	120.56 (13)	N1—C13—H13	118.6
C2—C3—H3	119.7	C12—C13—H13	118.6
C4—C3—C2	120.59 (14)	O3—C14—N2	121.84 (14)
C4—C3—H3	119.7	O3—C14—C10	119.46 (13)
C3—C4—C5	121.05 (14)	N2—C14—C10	118.71 (13)
			
O4—Mn1—O2—C1	−124.12 (16)	O2—C1—C2—C7	−169.99 (14)
O4^i^—Mn1—O2—C1	55.88 (16)	C1—C2—C3—C4	−178.83 (14)
N1^i^—Mn1—O2—C1	144.28 (16)	C7—C2—C3—C4	−0.8 (2)
N1—Mn1—O2—C1	−35.72 (16)	C1—C2—C7—C6	178.70 (15)
O2—Mn1—N1—C9	−23.31 (12)	C3—C2—C7—C6	0.6 (2)
O2^i^—Mn1—N1—C9	156.69 (12)	C5—C4—C3—C2	0.2 (2)
O2—Mn1—N1—C13	154.04 (12)	C3—C4—C5—C6	0.4 (2)
O2^i^—Mn1—N1—C13	−25.96 (12)	C3—C4—C5—C8	−179.60 (15)
O4—Mn1—N1—C9	62.76 (11)	C5—C6—C7—C2	0.0 (2)
O4^i^—Mn1—N1—C9	−117.24 (11)	C7—C6—C5—C4	−0.6 (2)
O4—Mn1—N1—C13	−119.89 (12)	C7—C6—C5—C8	179.46 (15)
O4^i^—Mn1—N1—C13	60.11 (12)	N1—C9—C10—C11	1.2 (2)
Mn1—O2—C1—O1	−26.6 (3)	N1—C9—C10—C14	−177.98 (13)
Mn1—O2—C1—C2	153.01 (12)	C12—C11—C10—C9	−0.1 (2)
Mn1—N1—C9—C10	176.18 (11)	C12—C11—C10—C14	179.01 (15)
C13—N1—C9—C10	−1.3 (2)	C10—C11—C12—C13	−0.8 (2)
Mn1—N1—C13—C12	−177.22 (13)	N1—C13—C12—C11	0.8 (3)
C9—N1—C13—C12	0.2 (2)	O3—C14—C10—C9	−9.9 (2)
O1—C1—C2—C3	−172.31 (14)	O3—C14—C10—C11	170.95 (15)
O1—C1—C2—C7	9.7 (2)	N2—C14—C10—C9	170.04 (14)
O2—C1—C2—C3	8.0 (2)	N2—C14—C10—C11	−9.1 (2)

Symmetry codes: (i) −*x*, −*y*, −*z*.

### Hydrogen-bond geometry (Å, °)

**Table:**

Cg1 and Cg2 are the centroids of the C2-C7 andN1/C9-C13 rings, respectively.

**Table:**

*D*—H···*A*	*D*—H	H···*A*	*D*···*A*	*D*—H···*A*
N2—H21···O1^ii^	0.88 (3)	1.97 (3)	2.8456 (19)	174 (2)
N2—H22···O3^iii^	0.86 (3)	2.09 (3)	2.952 (2)	172 (3)
O4—H42···O3^iv^	0.90 (3)	1.83 (3)	2.7071 (19)	164 (3)
C11—H11···O1^ii^	0.93	2.33	3.200 (2)	156
O4—H41···Cg1^v^	0.91 (2)	2.33 (2)	3.141 (2)	149 (3)
C4—H4···Cg2^v^	0.93	2.80	3.490 (2)	132

Symmetry codes: (ii) −*x*+1, −*y*−1, −*z*; (iii) −*x*+2, −*y*−1, −*z*; (iv) −*x*+1, −*y*, −*z*; (v) −*x*+1, −*y*+2, −*z*.
